# The Influence of Sporulation Conditions on the Spore Coat Protein Composition of *Bacillus subtilis* Spores

**DOI:** 10.3389/fmicb.2016.01636

**Published:** 2016-10-13

**Authors:** Wishwas R. Abhyankar, Kiki Kamphorst, Bhagyashree N. Swarge, Henk van Veen, Nicole N. van der Wel, Stanley Brul, Chris G. de Koster, Leo J. de Koning

**Affiliations:** ^1^Department of Mass Spectrometry of Bio-macromolecules, Swammerdam Institute for Life Sciences, University of AmsterdamAmsterdam, Netherlands; ^2^Department of Molecular Biology and Microbial Food Safety, Swammerdam Institute for Life Sciences, University of AmsterdamAmsterdam, Netherlands; ^3^Department of Cell Biology and Histology, Electron Microscopy Centre Amsterdam, Academic Medical CenterAmsterdam, Netherlands

**Keywords:** *Bacillus*, sporulation conditions, spores, proteomics, quantitative proteomics

## Abstract

Spores are of high interest to the food and health sectors because of their extreme resistance to harsh conditions, especially against heat. Earlier research has shown that spores prepared on solid agar plates have a higher heat resistance than those prepared under a liquid medium condition. It has also been shown that the more mature a spore is, the higher is its heat resistance most likely mediated, at least in part, by the progressive cross-linking of coat proteins. The current study for the first time assesses, at the proteomic level, the effect of two commonly used sporulation conditions on spore protein presence. ^14^N spores prepared on solid Schaeffer’s-glucose (SG) agar plates and ^15^N metabolically labeled spores prepared in shake flasks containing 3-(*N*-morpholino) propane sulfonic acid (MOPS) buffered defined liquid medium differ in their coat protein composition as revealed by LC-FT-MS/MS analyses. The former condition mimics the industrial settings while the latter conditions mimic the routine laboratory environment wherein spores are developed. As seen previously in many studies, the spores prepared on the solid agar plates show a higher thermal resistance than the spores prepared under liquid culture conditions. The ^14^N:^15^N isotopic ratio of the 1:1 mixture of the spore suspensions exposes that most of the identified inner coat and crust proteins are significantly more abundant while most of the outer coat proteins are significantly less abundant for the spores prepared on solid SG agar plates relative to the spores prepared in the liquid MOPS buffered defined medium. Sporulation condition-specific differences and variation in isotopic ratios between the tryptic peptides of expected cross-linked proteins suggest that the coat protein cross-linking may also be condition specific. Since the core dipicolinic acid content is found to be similar in both the spore populations, it appears that the difference in wet heat resistance is connected to the differences in the coat protein composition and assembly. Corroborating the proteomic analyses, electron microscopy analyses show a significantly thinner outer coat layer of the spores cultured on the solid agar medium.

## Introduction

Bacterial spores are of high interest because of their extreme resistance properties, especially toward nutritional and environmental stresses (Rose et al., 2007), and the heterogeneity amongst them. Due to their resistant nature, the methods to prevent bacterial contamination do not eliminate spores easily. This is not only a concern to the food processing and production industries but also to the cleaning rooms of industries as well as the medical sectors (Checinska et al., 2015). To develop spore prevention methods, the mechanisms of development and resistance of spores have to be understood completely. For a long time, the heat resistance of spores is of high interest in microbiology and therefore this property has been highly examined (Mundra et al., 2014). Stress resistant spores can germinate to form vegetative cells when the conditions become favorable resulting in food spoilage or sometimes food intoxication (Checinska et al., 2015). The spore germination behavior is heterogeneous and therefore an additional challenge to the food industries. The multiple layers of spores all contribute in some way to their resistance properties (Driks, 2002). It is believed that next to the dehydrated core, the cortex and coat contribute to the spores’ heat resistance. The core consists of the cell’s DNA, ribosomes, dipicolinic acid (DPA), and small acid-soluble proteins or SASPs (Setlow, 1988). The SASPs protect the DNA, while DPA forms a chelate complex with cations like calcium (Checinska et al., 2015). These chelate complexes are suggested to play a role in dehydration of the core, which in turn protects the spore from heat stress and UV light (Setlow et al., 2006). The major function of the external coat layers is to protect the spore from any enzyme or chemical assault and preserve the genome (Mckenney et al., 2013). A peculiarity of these proteinaceous layers is that ∼30% of it is said to be composed of insoluble cross-linked proteins.

The resistance of spores is affected by more than one factor during their development. The group of Sanchez-Salas et al. (2011) compared the wet heat resistance of young spores with the total produced (matured) spores from the same culture and found young spores to have a significantly lower wet heat resistance. Sanchez-Salas et al. (2011) concluded that changes in the spore coat during maturation are responsible for the higher wet heat resistance. Furthermore, a recent study from our group suggested that the inter-protein cross-linking might contribute to the heat resistance of the spores (Abhyankar et al., 2015). However, the influence of initial sporulation conditions on spore resistance properties was here not explicitly assessed. In a previous study, it had been shown that spores made at higher temperatures had a higher wet heat resistant than spores generated at lower sporulation temperatures (Melly et al., 2002). In addition, effects of the sporulation medium on spore’s stress resistance properties have been assessed in a number of studies. Dadd et al. (1983) showed that the level of nutrients and the amount of available divalent cations affect spore thermal stress resistance properties (Dadd et al., 1983). Rose et al. (2007) performed a study comparing the wet heat resistance of *Bacillus subtilis* spores prepared in 2x Schaeffer’s-glucose (2x SG) liquid and solid medium. The spores made on plates were more resistant to a temperature of 90°C in water than the spores made in liquid medium (Rose et al., 2007). The same group also concluded that there was no difference in the core water, DPA levels between two spore populations but these populations differed in their germination behavior. Yet, the effect of sporulation conditions on the spore’s (coat) proteome remains elusive.

The current research has focused on three main questions – (1) What is the influence of different sporulation conditions used in industries and research laboratories on the spore coat protein composition? (2) What is the influence of different sporulation conditions on spore coat inter-protein cross-linking and coat ultrastructure? (3) Does the (sporulation) condition-induced specific changes in spore thermal resistance, correlate with the changes in the spore coat proteome and changes in the extent of cross-linking of proteins such as CotG, CotU, GerQ, etc.? To answer these questions a quantitative proteomic analysis of *B. subtilis* spores made in liquid and on agar plates has been set-up under two commonly used sporulation conditions using two of the most common sporulation media. To that end ^14^N and metabolically labeled ^15^N vegetative cells are sporulated on ^14^N 2x SG agar solid plates or in a ^15^N-labeled 3-(*N*-morpholino) propane sulfonic acid (MOPS) buffered defined liquid medium, respectively. The former condition is generally used in industry as a condition known to yield most stress resistant spores and one that bears similarity to the conditions in the food chain. The latter condition is commonly used in fundamental microbiology laboratories as it allows for the dissection of the effects of individual medium components as well as provides homogenous conditions to maximize spore homogeneity useful for numerous biochemical studies. The spore suspensions are mixed 1:1 based on the optical density values and the protein ratios for the two different sporulation conditions are determined as the ^14^N: ^15^N protein abundance ratios with LC-FTMS analyses. DPA contents of two spore populations are determined to check the effect of each sporulation condition on the DPA content. Finally, to support our proteomics data, electron microscopy (EM) has been used and the thickness of different spore layers is measured. It appears that the spore coat protein composition of the two spore populations differs significantly. Especially, the outer coat proteome is affected by the sporulation conditions. The EM analyses show that the spores developed on the solid medium possess thinner outer coats compared to the spores formed in the liquid sporulation medium. Though the DPA levels of both spore populations are similar, the spores formed on plates are significantly more heat resistant. Interestingly, the crust proteins and certain coat proteins known to be involved in cross-linking in the spores are identified in higher abundance in the spores developed on solid medium relative to those developed in liquid medium. For these proteins, variations in peptide ratios are seen. This suggests that the enhanced heat resistance is possibly linked to the specific cross-linked coat proteins and the degree of cross-linking therein.

## Materials and Methods

### Bacterial Strain and Sporulation Conditions

**FIGURE 1:**
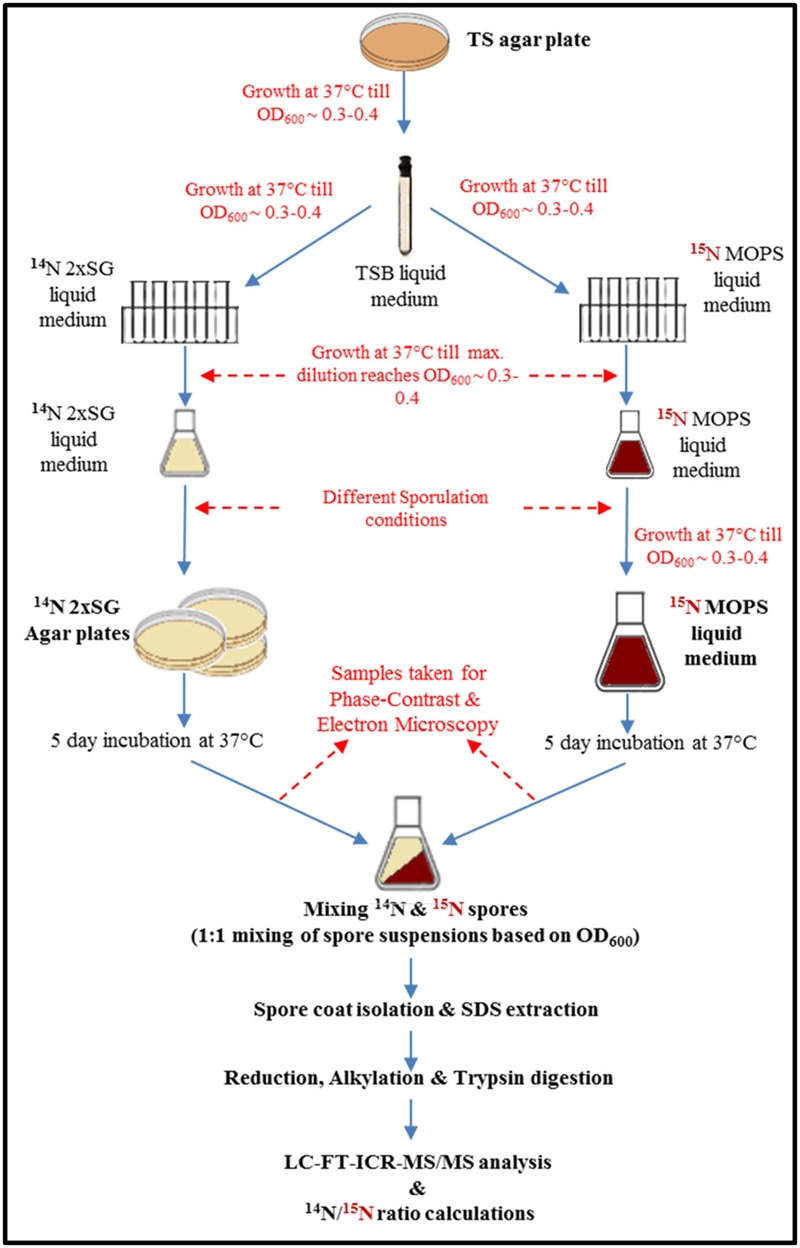
**Workflow of quantitative proteomics of spores prepared under the most common sporulation conditions used in industries (solid agar plates) and in routine microbiology laboratories (liquid medium).** The magenta color indicates presence of ^15^N in to the medium, cells, and therefore also in spores.

The *B. subtilis* wild-type strain PY79 used in this work was obtained from Dr. Eichenberger’s lab (New York University, USA). The aim was to study the effect of two of the most common yet drastically different sporulation conditions used in practice in industries and fundamental studies, respectively. Therefore we chose 2x SG agar as a solid medium and liquid defined medium buffered with MOPS in our study. Initially, the cells were cultured on a Tryptic Soy agar plate and incubated overnight at 37°C. A single colony was then inoculated in the liquid Tryptic Soy broth medium and cultivated until early exponential phase. The exponentially growing cells were then subjected to growth in serial dilutions (as shown in **Figure 1**) of 2x SG liquid medium (^14^N medium; further referred to as ^14^N cells) and a defined medium, buffered with MOPS supplemented with ^15^NH_4_Cl (further referred to as ^15^N cells). Further, the culture enrichment was done in 20 ml 2x SG and MOPS liquid media, respectively. For sporulation, the conditions differed significantly. The enriched ^14^N cells (0.1 ml) were spread on 10 plates of 2x SG agar (incubated at 37°C) whereas the enriched ^15^N cells were inoculated (1% final volume) to 500 ml of MOPS medium (incubated at 37°C at 200 rpm). The cells were allowed to sporulate for 5 days when the sporulation was induced by glucose exhaustion as described elsewhere (Abhyankar et al., 2011). Four independent biological replicates were prepared and analyzed.

### Spore Harvesting

After 5 days of incubation, the ^14^N spores were collected by scraping them off from the 2x SG agar plates and centrifugation whereas the ^15^N spores were harvested by centrifugation and removal of the supernatant. Both sets of spores were further purified as described in the literature (Abhyankar et al., 2011). In short, the spores were washed once with 0.01% Tween to kill the vegetative cells and further washed seven times with Milli-Q water. Any remaining vegetative cells were removed using a Histodenz gradient centrifugation, if needed as described elsewhere (Abhyankar et al., 2015). The yield of ^14^N spores was lower (10^9^spores/mL) compared to the ^15^N spores (10^11^spores/mL). The purified spore crops contained >99% phase bright spores.

### Mixing of ^14^N and ^15^N- Labeled Spores and Spore Coat Isolation

The harvested ^14^N and ^15^N spores were mixed in 1:1 ratio based on OD_600_ and re-suspended in cold 10mM Tris-HCl (pH 7.5). Subsequently the spores were disintegrated with 0.1-mm Zirconia Silica beads in a Bio-Savant Fast Prep 120 machine (Qbiogene, Carlsbad, CA, USA). A program of nine Fast-prepping rounds was set. One round lasted for 40 s at a maximum speed of 6. After every three rounds, the samples were placed on ice for 5 min to prevent protein degradation by over-heating. To remove non-covalently linked proteins and intracellular contaminants, isolated layers of insoluble spore material were washed extensively with 1 M NaCl and thermally treated for 10 min at 80°C in a water bath, with 50 mM Tris-HCl, pH 7.8 containing 2% SDS, 100 mM Na-EDTA, 150 mM NaCl, and 100 mM β-mercaptoethanol (Abhyankar et al., 2011). SDS-treated coats were washed four times with Milli-Q water and freeze dried overnight for immediate use for mass spectrometric (MS) analysis.

### Sample Preparation for MS Analysis

The freeze-dried coat material was subjected to reduction with 10 mM dithiothreitol in 100 mM NH_4_HCO_3_ (1 h at 55°C). To avoid the re-formation of the di-sulfide bridges, alkylation of the reduced coat material was performed with 55 mM iodoacetamide in 100 mM NH_4_HCO_3_ for 45 min at room temperature in the dark. Subsequently the proteins were digested for 18 h at 37°C with trypsin (Trypsin Gold Promega, Madison, WI, USA) using a 1:60 (w/w) protease: protein ratio. After 18 h, the tryptic digests were desalted using Omix μC18 pipette tips (80 μg capacity, Varian, Palo Alto, CA, USA) according to the manufacturer’s instructions. The peptides were collected in 25 μL 50% acetonitrile (ACN), 0.1% trifluoroacetic acid (TFA), and stored at -80°C (Abhyankar et al., 2011). Before analysis a fraction of eluted peptide material was freeze-dried and concentrated in 10 μL of 0.1% TFA and peptide concentration was measured at 205 nm (Scopes, 1974) with a Nanodrop ND1000 spectrophotometer (Isogen Life Sciences, De Meern, The Netherlands).

### Liquid Chromatography-Fourier Transform Ion Cyclotron Tandem Mass Spectrometry (LC-FT-ICR MS/MS) Analysis

LC-MS/MS data were acquired with an Bruker ApexUltra Fourier Transform Ion Cyclotron Resonance Mass Spectrometer (Bruker Daltonics, Bremen, Germany) equipped with a 7T magnet and a nano-electrospray Apollo II DualSource^TM^ coupled to an Ultimate 3000 (Dionex, Sunnyvale, CA, USA) High-Performance Liquid Chromatography system. For each digest, samples containing up to 200 ng of tryptic peptides were injected as a 20 μl 0.1%TFA, 3% ACN aqueous solution, and loaded onto a PepMap100 C18 (5 μm particle size, 100 Å pore size, 300 μm inner diameter × 5 mm length) precolumn. Following injection, the peptides were eluted via an Acclaim PepMap 100 C18 (3 μm particle size, 100 Å pore size, 75 μm inner diameter × 500 mm length.) analytical column (Thermo Scientific, Etten-Leur, The Netherlands) to the nano-electrospray source. LC gradient profiles of up to 120 min were used with 0.1% formic acid/99.9% H_2_O (A) and 0.1% formic acid/80% ACN/19.9% H_2_O (B) at a flow rate of 300 nL/min at a column temperature of 60°C. The gradient profiles were as follows 0 min 97%A/3%B, 2 min 94%A/6%B, 110 min 70%A/30%B, 120 min 60%A/40%B, 125 min 100%B. Data dependent Q-selected peptide ions were fragmented in the hexapole collision cell at an Argon pressure of 6 × 10^-6^ mbar (measured at the ion gage) and the fragment ions were detected in the ICR cell at a resolution of up to 60,000. In the MS/MS duty cycle, three different precursor peptide ions were selected from each survey MS. The MS/MS duty cycle time for one survey MS and three MS/MS acquisitions was about 2 s. Instrument mass calibration was better than 2 ppm over an m/z range of 250–1500. Raw FT-MS/MS data were processed with the MASCOT DISTILLER program, version 2.4.3.1 (64 bits), MDRO 2.4.3.0 (MATRIX science, London, UK), including the Search toolbox and the Quantification toolbox. Peak-picking for both MS and MS/MS spectra were optimized for the mass resolution of up to 60,000. Peaks were fitted to a simulated isotope distribution with a correlation threshold of 0.7, with minimum signal to noise of 2. The processed data, from the four independent biological replicates, were searched with the MASCOT server program 2.3.02 (MATRIX science, London, UK) against a complete *B. subtilis* 168 ORF translation database (Uniprot 2011 update, downloaded from http://www.uniprot.org/uniprot) combined with a protein contamination data base (compiled by and downloaded from Max Planck Institute of Biochemistry, Martinsried). Quantification using ^15^N-Metabolic [MD] labeling was included. Trypsin was used as enzyme and one missed cleavage was allowed. Carbamidomethylation of cysteine was used as a fixed modification and oxidation of methionine as a variable modification. The peptide mass tolerance was set to 25 ppm and the peptide fragment mass tolerance was set to 0.03 Dalton. MASCOT MudPIT peptide identification score was set to a cut-off of 18. The MASCOT protein identification reports were exported as XML and then imported in custom made Visual Basic (VBA) software program Protein Browser running in Microsoft Excel. The program facilitates organization and data mining of large sets of proteomics data. The identified proteins for each of the four replica of ^14^N/^15^N spore mixture coat isolate samples are listed in Supplementary Table S1A, together with their protein MASCOT score and number of peptide spectrum matches. These proteins were identified in at least two out of four replicates with at least two unique peptides. Using the quantification toolbox, the isotopic ratios for a subset of identified proteins were determined as an average of the isotopic ratios of the corresponding light over heavy tryptic peptides. This subset represents a selection of known and putative spore coat proteins, based on literature, sigma regulations, and localization studies. Critical settings were: require bold red: on, significance threshold: 0.05: Protocol type: precursor; Correction: Element ^15^N; Value 99.6; Report ratio L/H; Integration method: Simpson’s integration method; Integration source: survey; Allow elution time shift: on; Elution time delta: 20 s; Std. Err. Threshold: 0.15, Correlation Threshold (Isotopic distribution fit): 0.98; XIC threshold: 0.1; All charge states: on; Max XIC width: 200 s; Threshold type: at least homology; Peptide threshold value: 0.05; unique pepseq: on. All reported isotopic peptide ratios were manually validated by inspection of the spectral MS data. The MASCOT DISTILLER protein quantification reports were exported in excel format and then imported in the Protein Browser program. To correct for possible errors in ^14^N/^15^N 1:1 culture mixing, the ^14^N/^15^N ratios were normalized on the median for each replicate dataset. For quantification a sub-selection of putative and known coat proteins was made based on previous literature (Abhyankar et al., 2015), localization studies and their regulating sigma factors. The quantified putative coat proteins for each of the four replica ^14^N/^15^N spore mixture coat isolate samples are included in Supplementary Table S1B, together with their protein MASCOT score and light over heavy (L/H) isotopic ratio. The proteins that were quantified at least in two of the four replicates have been included in the results. The proteomics data has been deposited to the ProteomeXchange Consortium (Vizcaíno et al., 2014) via the PRIDE partner repository with the dataset identifier PXD004473.

### Measurement of Thermal Resistance of ^14^N and ^15^N Spores

One milliliter of spores (^14^N as well as ^15^N spores) with an OD_600_ of 2 were heat activated (70°C, 30 min) in a water bath. The vegetative cells, if any, were inactivated by the heat activation. After heat activation the 1 ml of spores were injected with a syringe into metal screw-cap tubes with 9 ml of sterile Milli-Q water which were pre-heated for 20 min in a glycerol bath (85°C). The spore suspensions from three biological replicates were heated for 10 min (85°C) in the glycerol bath inducing thermal stress. After 10 min, the tubes were cooled on ice. One milliliter of the spore suspension was serial diluted in sterile Milli-Q water and 100 μl of sample from last three dilutions was spread on Tryptic Soy agar plates. The heat resistance was determined by counting the number of colonies after 48 h incubation at 37°C (Kooiman et al., 1973). Two technical replicates were performed for each biological replicate.

### Measurement of DPA Concentration in ^14^N and ^15^N Spores

Dipicolinic acid analysis was performed according to an established protocol (Janssen et al., 1958). Spore suspension with a fixed OD_600_ was freeze dried and weighed. Consequently, the spores were suspended in 5 ml of sterile Milli-Q water and autoclaved for 15 min at 121°C. The spores were then cooled on ice, acidified with 100 μl of 1 N acetic acid and incubated for 1 h at room temperature. After 1 h, the spores were centrifuged (10 min at 1500 × *g*) and 4 ml of the supernatant was transferred to new test tube. One milliliter of the color reagent [1 % Fe(NH_4_)_2_(SO_4_)_2_.6H_2_O along with 1 % ascorbic acid in 0.5 M acetate buffer of pH 5.5] was added to the supernatant. By measuring the A_440_, a standard curve was prepared for the concentration range of 20–140 μg/ml DPA. The A_440_ of the mixture was measured and the amounts were calculated from the standard curve.

### Electron Microscopy

For each sporulation condition, the spores were fixed in 1% glutaraldehyde and 4% paraformaldehyde in 0.1 M Phosphate buffer (pH 7.4) for 60 min After fixation the spores were washed in distilled water, block stained overnight in 1.5% uranyl acetate to enhance contrast in the electron microscope, washed in distilled water, and osmicated for 60 min in 1% OsO_4_ in water (Electron Microscopy Sciences, Hatfield, PA, USA). Subsequently, the spores were dehydrated in an alcohol series (of ethanol) and embedded into propylene oxide/Epon 1:1 before embedding into LX-112 resin (Ladd Research, Williston, VT, USA). Between each step, the spores were centrifuged down. After polymerization at 60°C the Epon blocks with the spores were prepared for ultrathin sectioning. Ultrathin sections of 90 nm were cut on a Reichert EM UC6 with a diamond knife, collected on Formvar coated grids and stained with uranyl acetate and lead citrate. Sections were examined with a FEI Technai-12 G2 Spirit Biotwin transmission electron microscope (TEM). Images were taken with a Veleta camera (Olympus, SIS). For measurement of the thickness of the coat layers, using the iTEM software (Emsis) the images of cross-sectioned spores were taken at a magnification of 120,000×. For these measurements, 80 spores per replicate were used.

## Results

### Differences in Coat Protein Levels of Spores Developed under Solid and Liquid Medium Conditions

**FIGURE 2:**
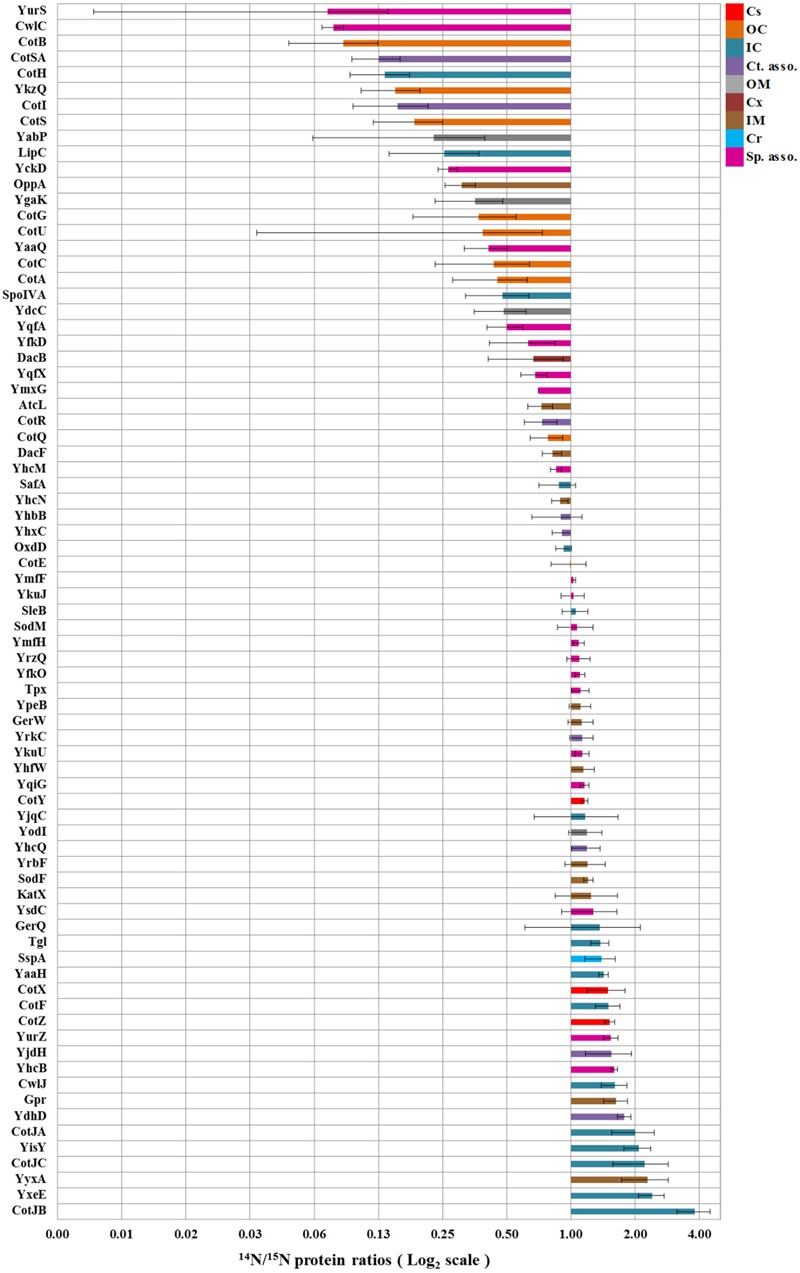
**Fold changes in spore coat protein expression in spores cultured on solid agar plates and in shake flask with liquid medium.** The bars indicate the mean ratios of proteins across four replicates while error bars indicate the standard deviations in these ratios. By definition, the values are within 95.40% confidence interval in the range of the error bars. The protein localization is based upon their sigma factor regulations and the available literature (van Ooij et al., 2004; Imamura et al., 2010; McKenney et al., 2010; Driks and Eichenberger, 2016). Cs, Crust; OC, Outer coat; IC, Inner coat; Ct. asso., Spore coat associated proteins; OM, Outer membrane; Cx, Cortex; IM, Inner membrane; Cr, Core; Sp. asso., Spore associated proteins.

A total of 351 proteins have been identified across four replicates (Supplementary Table S1A). Because part of the identified proteins are co-isolated cytosolic proteins, only a selected group of spore proteins have been quantified by calculating their ^14^N/^15^N isotopic ratio (Supplementary Table S1B). This sub-selection includes outer coat, inner coat, and crust proteins as identified based on their sigma factor regulation and/or localization (Abhyankar et al., 2011). The mean average of light/heavy ratios of the proteins over the four replicates are plotted on a Log_2_ scale (**Figure 2**) as coat protein fold changes between the spores cultured under solid and in liquid medium conditions. The error bars indicate the standard deviation per protein. The results in **Figure 2** show that most of the outer coat proteins such as CotA, CotG, CotC, CotU, etc., are lower in abundance in spores developed on solid sporulation medium (represented in the upper part of the figure with isotopic ratios lower than 1). Also all known crust proteins CotX, CotY, and CotZ as well as many of the inner coat proteins such as CotJA, CotJB, etc., are seen to be highly abundant in the spores obtained from solid sporulation media (represented in the lower part of the figure with isotopic ratios greater than 1).

### Thermal Resistance of ^14^N and ^15^N Spores Obtained from SG Plates and MOPS in Shake Flask Conditions

**FIGURE 3:**
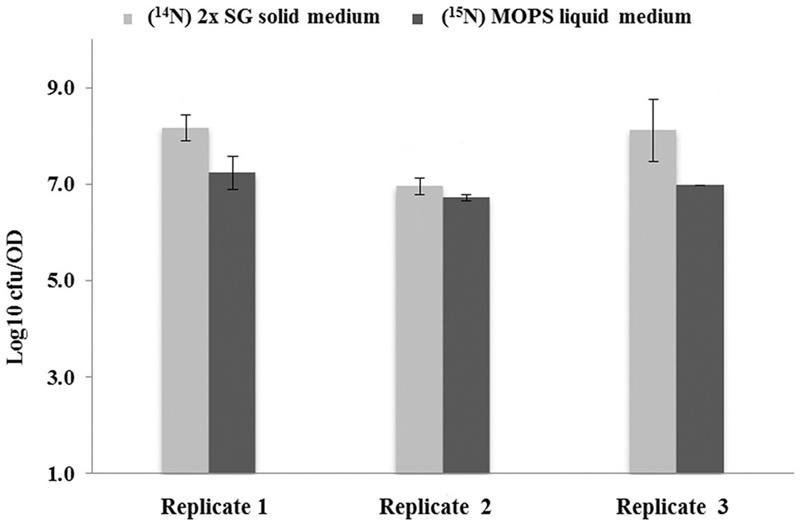
**Thermal resistance of spores developed under different sporulation conditions.** Log_10_ values of colony forming units (CFU) obtained after 48 h incubation per OD_600_ of spores. The values represent the mean averages for three biological replicates where the error bars represent the standard deviation observed for two technical replicates. By definition, the values are within 95.40% confidence interval in the range of the error bars.

The results for the thermal resistance test for spores at 85°C for 10 min are shown in **Figure 3**. The data shows the Log_10_ number of colonies, grown on the TSA plates 48 h after the thermally induced stress had been given. Shown are mean values, averaged over two technical replicates performed for three biological replicates. It appears that the spores prepared on the 2x SG solid medium plates yield significantly more colonies thereby indicating a higher thermal resistance than observed for the spores generated under liquid medium condition.

### The Core DPA Content of ^14^N and ^15^N Spores Obtained from SG Plates and MOPS in Shake Flask Conditions

**FIGURE 4:**
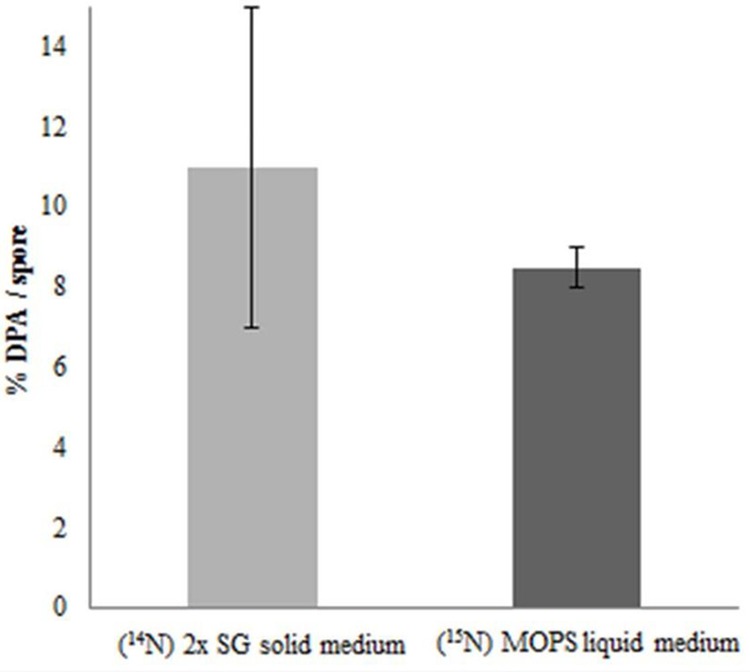
**Core dipicolinic acid (DPA) content as % per spore dry weight of spores developed under different sporulation conditions.** The error bars indicate the standard deviation within two technical replicates. By definition, the values are within 95.40% confidence interval in the range of the error bars.

In order to check the role of DPA on thermal resistance of spore cultured under different sporulation conditions, the DPA concentrations were measured. For both spore populations, the DPA content analyses show no significant differences. As shown in **Figure 4** the DPA amount, mean averaged over two technical replicate analyses in both spore populations, is between the previously reported range of 5–15% (by weight) per spore (Gerhardt and Marquis, 1989).

### Morphology of ^14^N and ^15^N Spores Obtained from SG Plates and MOPS in Shake Flask Conditions

**FIGURE 5:**
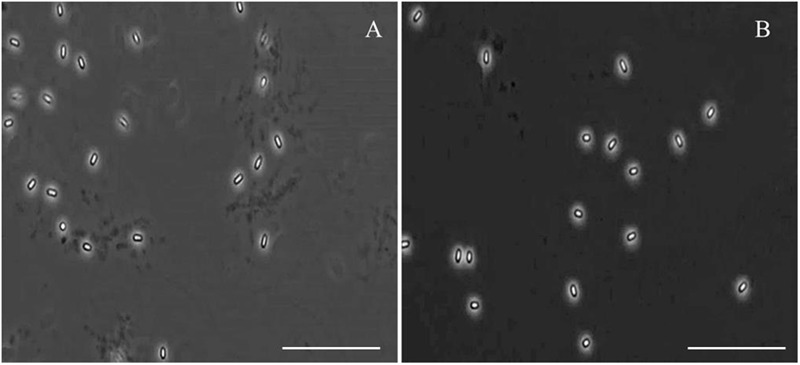
**Phase contrast microscopic images of spores cultured on solid agar plates **(A)** and in shake flasks containing liquid medium (B)**. The spores are observed under 100× magnification with oil under simple phase contrast microscope. Scale bars indicate 10 μm.

**FIGURE 6:**
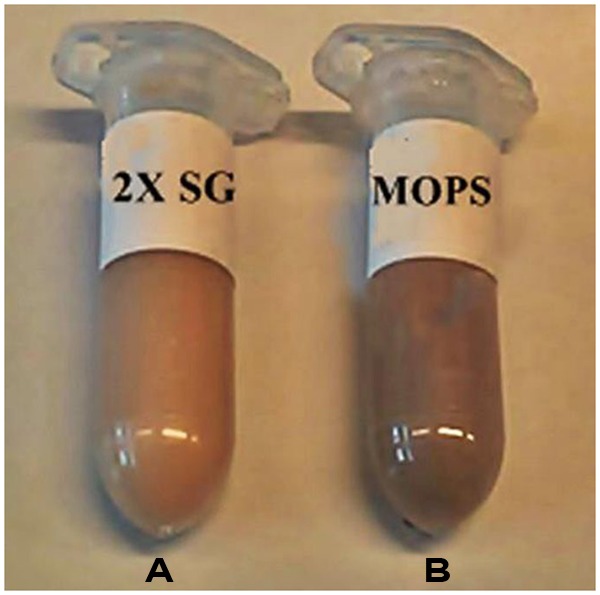
**Appearance of liquid suspensions of spores cultured on 2x SG agar plates **(A)** and in 3-(*N*-morpholino) propane sulfonic acid (MOPS) buffered defined liquid medium in shake flask (B)**. Two spore crops differ in their color as liquid suspensions in sterile Milli-Q water with equal OD_600_.

**FIGURE 7:**
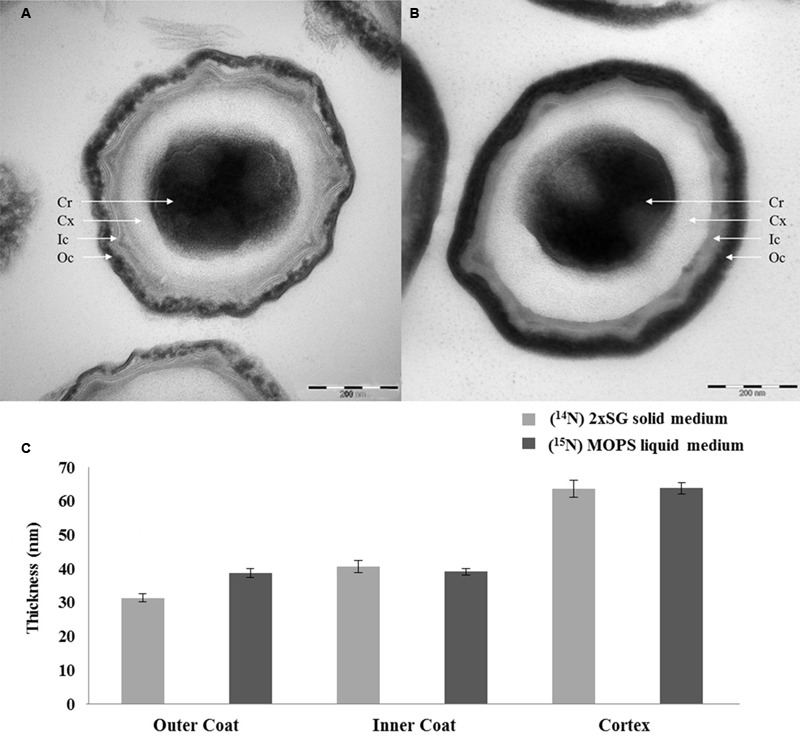
**Electron microscopic images of spores developed under solid **(A)** and liquid **(B)** medium conditions with the corresponding thickness of the spore coat layers (C)**. The coat layer thicknesses are averaged over 80 individual spores. The error bars indicate the standard deviations between two biological replicates. By definition, the values are within 95.40% confidence interval in the range of the error bars. Cr, core; Cx, cortex; Ic, inner coat; Oc, outer coat.

The spores prepared on solid and in liquid media under the phase contrast microscope show no significant morphological difference in terms of size and shape of both spore populations (**Figure 5**). However, the spores prepared on the 2x SG agar plates and those prepared in MOPS liquid medium in shake flasks show a clear color difference when seen as liquid suspensions of similar OD (**Figure 6**). The spores prepared on the 2x SG agar plates have a light brown/beige color, while spores prepared in the liquid MOPS medium are colored dark brown. The electron microscopic images of the spores (**Figure 7**) clearly show characteristic differences between the spores cultured under solid and liquid medium conditions. While the outer coat layer of the spores cultured under solid medium condition is significantly thinner, the thicknesses of the inner coat and the cortex layers are comparable to the corresponding layers in the spores prepared under liquid medium condition (**Figure 7**).

## Discussion

The protein fold changes depicted in **Figure 2** show that most of the inner coat and all the known crust morphogenetic proteins (CotX, CotY, and CotZ; Imamura et al., 2011; Driks and Eichenberger, 2016) are more abundant (^14^N:^15^N ratio > 1) and most of the outer coat proteins such as CotA, CotB, CotC, CotG, CotQ, and CotU (Driks and Eichenberger, 2016) are less abundant (^14^N:^15^N ratio < 1) in spores obtained from cultures grown on solid agar plates relative to the cells that were sporulated in liquid medium. Significantly, CotE levels are not affected. CotE is a pivotal protein coordinating the formation of the outer coat (Driks, 2002) and directing the assembly of a large set of inner and outer coat proteins (Kim et al., 2006). Different regions of CotE are responsible for the assembly and deposition of spore coat proteins. Proteins CotA, YaaH, CotR, CotG, CotB, CotS, and CotSA are dependent on the C-terminal of CotE for their deposition (Little and Driks, 2001). It is remarkable that most of these proteins are found to be less abundant in spores cultured on solid 2x SG agar plates. No tryptic peptide from C-terminal region of CotE has been identified in this study. This can be either due to the lack of lysine and arginine cleavage sites in this region or due to the involvement of the C-terminal in linking and depositing other proteins. Both CotJC and CotJB (∼17 kDa) are found to be significantly abundant in spores prepared on solid agar plates which is consistent with the report that CotJC and CotJA, which are part of the same operon as CotJB, directly influence each other (Kim et al., 2006).

In the current study, we compare the two well-known sporulation conditions, one used often in industries to generate high heat resistant spores on solid plates, and one in defined liquid medium that provides a basis for laboratory experiments that seek for the molecular mechanisms of spore resistance. Although, the phase contrast microscopic images in **Figure 5** show no significant differences in the morphology and spore size of the two spore crops, the spore suspensions in **Figure 6** show a more intense browning for the spores cultured in the shake flask, which agrees with the observed increased abundance of the outer coat protein CotA in those spores. This protein is known to produce the brown melanin-like pigment in spores (Hullo et al., 2001). These results are in contrast with those obtained by Rose et al.(2007) as they observed no major difference in the CotA levels but a large difference in pigment production, i.e., CotA activity when they compared two different sporulation conditions using the same medium. This indicates that the CotA activity and CotA levels might not always be comparable. This observation also calls for a detailed EM studies of these spore populations. Upon zooming in with an electron microscope, the images clearly show that the crust and outer coat layer, averaged over 80 spore images is about 10 nm thicker for the spores cultured in the liquid defined medium (**Figure 7**). This agrees with the quantitative proteomic results, which show high abundance for most of the crust and outer coat proteins in these spores. The EM images further show no significant differences between the thickness of the inner coat and cortex layers, while no significant difference has been found in the DPA content in the core of both spore crops (**Figure 4**). Also Rose et al. (2007) have found no significant difference in the core water levels of spores prepared in the same medium but with different sporulation conditions. The DPA content, linked to the thermal resistance of spores (Orsburn et al., 2008), is comparable in both ^14^N and ^15^N spores in our study and yet the thermal resistance of the spores cultured on the solid agar is significantly higher. For the coat proteins Tgl, GerQ, KatX (catalase), YjqC (catalase), SodM unusual large variations in the isotopic ratios have been found between the four replicates as shown by their large standard deviation (**Figure 2**). These proteins are suggested to be involved in inter-protein cross-linking during spore maturation (Driks, 1999; Ragkousi and Setlow, 2004; Checinska et al., 2015), and variations in peptide ratios suggest that cross-linking during maturation hampers the efficient digestion of these proteins (Abhyankar et al., 2015) and that the progress of cross-linking is critical to reproduce. Similarly, the cysteine rich crust proteins CotX, CotY, and CotZ are also assumed to be cross-linked via di-sulfide bonds. Yet, no large variations of protein isotopic ratios are observed for these proteins. This is because the di-sulfide links in the crust are cut during processing of the spores before the tryptic digestion. These proteins like the ones mentioned above are present at higher abundance in the spores cultured on the solid agar plates. This suggests that the enhanced heat resistance is likely to be linked to the sum of specific cross-linked coat proteins and the degree of cross-linking.

## Conclusion

It appears that the spores developed on solid 2x SG agar plates and those in liquid defined medium in a shake flask, have a distinct coat protein composition, which is correlated, and likely functionally connected, to the difference in thermal resistance. Despite the higher abundance of most of the outer coat proteins and consequently a thicker outer coat layer, the spores obtained from cells sporulated under liquid medium conditions have a lower thermal resistance. Coat and crust proteins typically involved in cross-linking are more abundant in the thermally resistant spores (prepared on agar plates) that possess thinner outer coats. With the DPA contents of both spore crops being comparable, the difference in thermal resistance could well be linked to the difference in the degree of cross-linking in the coat.

## Author Contributions

WA and LdK formulated the research concept, contributed equally to drafting of the manuscript, and performed data analysis. KK and BS performed the laboratory work. HvV and NvW carried out the electron microscopy measurements. SB and CdK contributed by guiding the laboratory work and by critically reading the manuscript.

## Conflict of Interest Statement

The authors declare that the research was conducted in the absence of any commercial or financial relationships that could be construed as a potential conflict of interest.
